# Dwarf shrub facilitates seedling recruitment and plant diversity in semiarid grasslands

**DOI:** 10.1371/journal.pone.0212058

**Published:** 2019-02-07

**Authors:** Sofía L. Gonzalez, Luciana Ghermandi

**Affiliations:** Laboratorio Ecotono, INIBIOMA, Universidad Nacional del Comahue, CONICET, Bariloche, Río Negro, Argentina; Centro de Edafologia y Biologia Aplicada del Segura, SPAIN

## Abstract

The facilitation mechanism maintains ecosystem richness by increasing seedling recruitment. Overgrazed grasslands of northwestern Patagonia are invaded by shrubs that could promote the seedling recruitment of forage species. We investigated the role of *Acaena splendens* shrubs on the maintenance of diversity and its usefulness as a nurse shrub in the recruitment of *Festuca pallescens*, a grass of high forage value present with a low cover in degraded grasslands. To test the performance of *A*.*splendens* as a nurse plant in non-degraded grassland, we recorded the species richness four years inside of *A*. *splendens* senescent shrubs and in gaps among dominant tussock grasses. Species were grouped in four functional groups: annual and biannual herbs and grasses, perennial herbs, perennial grasses and shrubs. To test the usefulness of *A*. *splendens* in the restoration of degraded grassland, we monitored the seedling emergence and survival of *F*. *pallescens* inside *A*. *splendens* and in gaps. We related seedling survival to meteorological and microenvironmental conditions. Species richness was higher in Acaena nurse plants than in gaps. The frequency of functional groups, with exception of annual and biannual herbs and grasses, were higher in Acaena than in gaps. Seedling emergence and survival of *F*. *pallescens* were higher in Acaena, but the seedlings died in summer in both microsites. Mean maximum temperature was higher and mean minimum humidity lower in gaps than in Acaena during spring. However, the spring-summer season in which we monitored *F*. *pallescens* survival, was exceptionally dry and hot, affecting the survival of *F*. *pallescens* seedlings. Our results show that *A*. *splendens* act as a nurse species increasing the richness in the non-degraded grassland and facilitating the seedling recruitment of an important forage species in the degraded grassland. Nevertheless, the facilitation mechanism will fail in drought conditions, indicating that this restoration tool is limited by climate.

## Introduction

Seedlings represent a critical stage of plant life cycles [1, 2], driving plant population dynamics [3]. Seedling recruitment depends on seed presence and the availability of microsites (safe sites, *sensu* [4]) that provide suitable conditions for seed germination and seedling establishment. These conditions include adequate temperature, light, soil moisture, and protection from herbivory [3]. Adult plants can provide safe sites where more vulnerable species can become established and grow [5]. This positive plant-plant interaction, called “nurse plant syndrome” *sensu* [6], is particularly common in harsh environments [7, 8].

The canopy of a nurse plant moderates high temperatures by decreasing the amount of radiation that reaches protected plants. As a consequence, air and soil temperature are lower, and soil water content may be is higher than in nearby bare areas [5]. Nurse plants also improve soil resources through litter accumulation [9, 5]. The resulting reduction in environmental stress accelerates the natural recovery of disturbed areas [10], and for this reason, the interest in using nurse plants as a tool in the passive restoration of degraded habitats has increased over the past two decades [11, 12].

The relationship between facilitation and competition is modulated by environmental conditions [13]. In arid and semiarid environments the facilitative effects of nurse plants on the establishment of other species can diminish or even collapse under an infrequent stress, such as an extreme drought occurring during growing season [14, 15]. In these circumstances water deficit promotes competition between plants, weakening the facilitation effects [16]. Understanding the relationships between water availability and plant-plant interactions in arid and semiarid regions will provide useful information for the implementation of restoration programs.

Biodiversity is essential to the functioning of natural ecosystems, as it has a strong influence on ecosystems in terms of nutrient cycling, productivity and resistance to invasion [17]. Identifying the processes that maintain biodiversity is a big challenge in community ecology [18]. Classical studies on this issue focused on negative interactions, such as competition, as regulators of biological diversity [19], but more recent studies have considered the importance of positive plant-plant interactions, such as the nurse effect [20, 21]. Nurse plants can increase species richness, improving local environmental conditions and enhancing species coexistence [22, 23]. However, some authors have not recorded any increase in species richness in association with nurse plants [24, 25].

Semiarid extra-Andean grasslands are dominated by *Festuca pallescens* (St. Ives) Parodi and *Pappostipa speciosa* (Trin & Rupr.) Romasch and are the most productive of northern Patagonia (Argentina). Since the early 1900s these species have been used for livestock breeding without taking sustainability into account [26]. When confined to fenced areas, livestock impose greater harvesting pressure on vegetation than native herbivores, promoting desertification [27]. Overgrazing causes a decrease in nutrients and an increase in erosion and soil compaction, affecting the availability of safe sites [28]. In Patagonia, overgrazing works in negative synergy with water stress [27]. Livestock management, such as cattle exclusion or restriction, is usually ineffective in severely grazed areas since the degraded grasslands have reached an undesirable steady state [29, 30]. When the threshold between healthy and degraded grassland is crossed, vegetation cover declines rapidly, affecting ecosystem multi-functionality [29]. Degraded ranches become economically unsustainable, and in many cases are even abandoned.

*Festuca pallescens* is a Patagonian cool-season grass with high forage value, and its recruitment is dependent on autumn and spring rains [31, 32]. Livestock grazing reduces *F*. *pallescens* cover, diminishing the forage quality of grassland [26] and promoting the advance of shrubs [33].

*Acaena splendens* Hook et. Arn (Rosaceae) is a native, unpalatable dwarf shrub present in Argentina and Chile [34], where it frequently colonizes sandy soils and degraded areas [35]; its abundance is generally related to overgrazing [33]. *Acaena splendens* reproduces only by thorny achenes, principally dispersed by animals [36]. Plants are 40–60 cm high and short-lived (approximately 20 years) [34]. The compact canopy of *A*. *splendens* dies centrifugally (from the center to the edge), producing an internal area that is colonized by seedlings of the same and other species [37]. The seedlings of the same species begin a self-replacement cycle also documented in *Calluna vulgaris* [38] and *Richea acerosa* [39], whereas the seedling recruitment of other species could maintain or increase grassland diversity.

The aim of this work was to study the facilitation performance of *A*. *splendens* with respect to the promotion of grassland diversity, and its usefulness in the restoration of degraded areas. We monitored species richness, seedling recruitment and seedling survival for seven years in two microsites: senescent plants of *A*. *splendens* and gaps among the dominant tussock grasses. To explain the facilitation mechanism, the soil and microclimatic variables were analyzed in both microsites and the effect of seasonal precipitation on seedling recruitment was interpreted. To test the performance of *A*. *splendens* as a nurse plant for *F*. *pallescens*, seeds of this species were sown in senescent plants of *A*. *splendens* and in adjacent bare soil, and *F*. *pallescens* seedling survival was monitored. We hypothesized that *A*. *splendens*: increases grassland diversity, propitiates self-replacement, and improves *F*. *pallescens* recruitment.

## Materials and methods

### Study site

The grasslands where we carried out the study are located in northwestern Patagonia (San Ramón Ranch, 41°03´19´´S and 18°01´50´´W, Argentina). The climate regime is of Mediterranean type, with 60% of the precipitation accumulating in autumn-winter [40]. Mean annual precipitation is 582 mm and mean annual temperature is 9°C (San Ramón Ranch Meteorological Station 1929–2017, unpublished data). Strong west-northwestern winds blow throughout the year, accentuating water stress [41]. Soils are Haploxerolls characterized by sandy loam texture and moderate organic matter content [42].

The vegetation is dominated by *F*. *pallescens* and *Pappostipa speciosa* tussock grasses, the former being preferred by livestock [43]. *Acaena splendens* (Rosaceae) shrubs are present in different proportions depending on the grassland degradation. The study area includes grassland in healthy condition (henceforth “non-degraded grassland”) with a total vegetation cover of 73%, of which 5% is *A*. *splendens*, and overgrazed grassland (henceforth “degraded grassland”) with a total vegetation cover of 47%, of which 30% is *A*. *splendens* cover.

### Sampling design

The species richness of the non-degraded grassland has been recorded in the context of a long-term monitoring project (1999–2018, unpublished data). In this grassland, in November 1999 we selected 10 senescent plants (henceforth “Acaena-ND”), and 18 areas of gaps with herbaceous species and seedlings of shrubs and tussock grasses, each measuring 1 ± 0.3 m^2^ (henceforth “Gap-ND”). Both microsites were used to test the facilitation performance of *A*. *splendens* shrubs on species richness. *Acaena splendens* senescent plants maintain a live ring that defines an inner area of 1± 0.4 m^2^. Fortunately, the dead *A*. *splendens* areas and the areas of gaps are similar in size, allowing comparison of species richness. We recorded the seedlings of all species in both microsites in November (spring) of 1999, 2000, 2001 and 2005. *Acaena splendens* establishment was monitored in November of 1999, 2000, 2001, 2005 and 2006, whereas *A*. *splendens* seedling mortality was recorded in February-April (late summer and fall) of 2002, 2003, 2004, and 2005.

In the degraded grassland, in January 2015 (summer), we labeled 20 senescent *A*. *splendens* plants, called “Acaena-D”, and 20 gaps microsites called “Gap-D”. We harvested *F*. *pallescens* seeds from populations near the degraded areas to conserve the gene pool. Seeds with a healthy appearance were selected using the pressure method [44]. In April 2015 (fall) we gathered 20 soil samples from each microsite (20 cm in diameter and 5 cm in depth). The soil samples were sieved to remove any *F*. *pallescens* seeds and were carried again to the microsites where samples had been collected. We sowed 40 *F*. *pallescens* seeds at 1 cm depth in each microsite, and excluded herbivory by exotic hares and native rodents by means of wire enclosures. We recorded seed germination and monitored and counted the leaves on tagged seedlings in May, September and December 2015, and April 2016.

To determine and compare microclimate conditions we recorded temperature (°C) and relative humidity (%) every hour, from September to December 2015 (spring) with dataloggers (Cavadevices) in both microsites. Each datalogger had two sensors: one to record temperature and one to record relative humidity.

To characterize and compare the soil nutrients we collected 10 soil samples of 5 cm depth (500 mg each) in each microsite in April 2016. Soil samples were stove dried at 105°C until constant weight and stored in a desiccator. Total soil C and N content was determined with a CN analyzer, with combustion at 900°C (Flash EA 1112 Series Thermo Electron Corporation).

### Data analysis

We analyzed the data with non-parametric tests since the data violated normality and homoscedasticity assumptions, even after logarithmic (*x*+1), square root, or arcsine transformations.

Species richness for the non-degraded grassland was calculated from the data collected during the long-term monitoring project (1999–2018, unpublished data). In the same grassland we calculated the number of species present in Acaena-ND and Gap-ND microsites for each year (microsite species richness). We compared richness between Acaena-ND and Gap-ND microsites using the non-parametric Mann-Whitney test. Richness and *A*. *splendens* seedling recruitment at each microsite were compared between years using non-parametric Friedman Anova analysis, and a posteriori pairwise comparisons were made with the Wilcoxon test.

Species in Acaena-ND and Gap-ND microsites were grouped into four functional groups: annual grasses and annual and biannual herbs, perennial herbs, perennial grasses, and shrubs. We calculated the mean frequency of species of each functional group for each microsite and year. Mean frequency of functional groups was compared for the same microsite using the non-parametric Kruskal-Wallis test, and between microsites using the Mann-Whitney test. A posteriori p-value adjustment was applied with the Bonferroni test.

We compared the *F*. *pallescens* seedling number between Acaena-D and Gap-D microsites for each sampling date with the non-parametric Wilcoxon test.

We compared the mean daily temperature and humidity (calculated from the daily records) among September, October, November, and December. The replications of each variable were 30 or 31 depending on the number of days of the month. The daily temperature and air humidity data for both microsites in the degraded grassland were used to calculate mean maximum monthly temperature, and mean minimum monthly humidity percentage. We used t-tests to compare these data after they were transformed (log 10) to achieve normality and homocedasticity. To calculate the frequency of maximum temperature and minimum humidity we used five class intervals for temperature (0-10C°; 10-20C°; 20–30°C; 30–40°C; and 40–50°C) and seven class intervals for humidity (0–10%; 10–20%; 20–30%; 30–40%; 40–50%; 50–70%; 70–100%). We used chi-squared analysis to assess whether the frequencies of maximum temperature and minimum relative humidity values were similar for Acaena-D and Gap-D microsites in each month.

We used a t-test for one sample to compare values of accumulated precipitation and mean temperature with mean historical values of precipitation (1928–2016) and temperature (1970–2016) (San Ramón ranch meteorological station 1929–2016, L. Ghermandi unpublished data) in order to relate weather conditions to: 1- the richness of Acaena-ND and Gap-ND microsites (1999, 2000, 2001 and 2005) in the non-degraded grassland, 2- seedling recruitment of *A*. *splendens* in the non-degraded grassland (1999, 2000, 2001, 2005 and 2006), and 3- seedling recruitment of *F*. *pallescens* in the degraded grassland (2015 and 2016).

## Results

### Meteorological conditions

The springs of 1999 and 2001 were dry (1999: *t*_*87*_ = 6.99, *P* < 0.001; 2001: *t*_*87*_ = 10.52, *P* < 0.001) and hot (1999: *t*_*47*_ = -14.97, *P* < 0.001; 2001: *t*_*47*_ = -6.01, *P* < 0.001), whereas the springs of 2000 and 2005 were wet (2000: *t*_*87*_ = -10.18, *P* < 0.001; 2005: *t*_*87*_ = -3.4, *P* = 0.001) (Table 1). Precipitation in August 1999 was over twice the historical value (*t*_*87*_ = -21.13, *P* = 0.001) and the temperature was also higher than the historical value (*t*_*47*_ = 7.2, *P* < 0.001) (Table 1). In August 2005, precipitation was significantly higher than the historical value (*t*_*87*_ = 8.6, *P* < 0.001) but the temperature was lower (*t*_*47*_ = -3.9, *P* < 0.001). August 2000 and 2001 were hotter than the historical value (*t*_*47*_ = -7.8, *P* < 0.001) and were dry (2000: *t*_*87*_ = -5.17, *P* < 0.001; 2001:*t*_*87*_ = 9.13, *P* < 0.001) (Table 1).

**Table 1:** Precipitation and temperature in spring (1998–2001, and 2005) and August (1999–2001, 2005 and 2006). Comparisons between the accumulated precipitations (Pp) of spring (sep-oct-nov) and August, the mean temperature (T) of the same periods, and the mean historical values ± SD (San Ramón ranch meterorological station, L. Ghermandi unpublished data).

Year	Pp (mm)	T (°C)
**Spring**		
1998	<50.8***	>8.8***
1999	<54.9***	>9.9***
2000	>145.8***	<8*
2001	<36.1***	>8.9***
2005	>110.4***	<7.8***
Historical	91.9±11.6	8.2±0.5
**August**		
1999	>181.3***	>5***
2000	<49.8***	>5.1***
2001	<30***	>5.1***
2005	>95.6***	<2.2***
2006	69.2 n.s.	3.4 n.s.
Historical	75.4±47.6	3.7±1.2

“<“indicates that the value of Pp or T is lower than the historical value whereas “>” indicates that these values are higher than the historical value.

*P<0.05

**P<0.01

***P<0.001, ns = non significant

**Fig 1:**
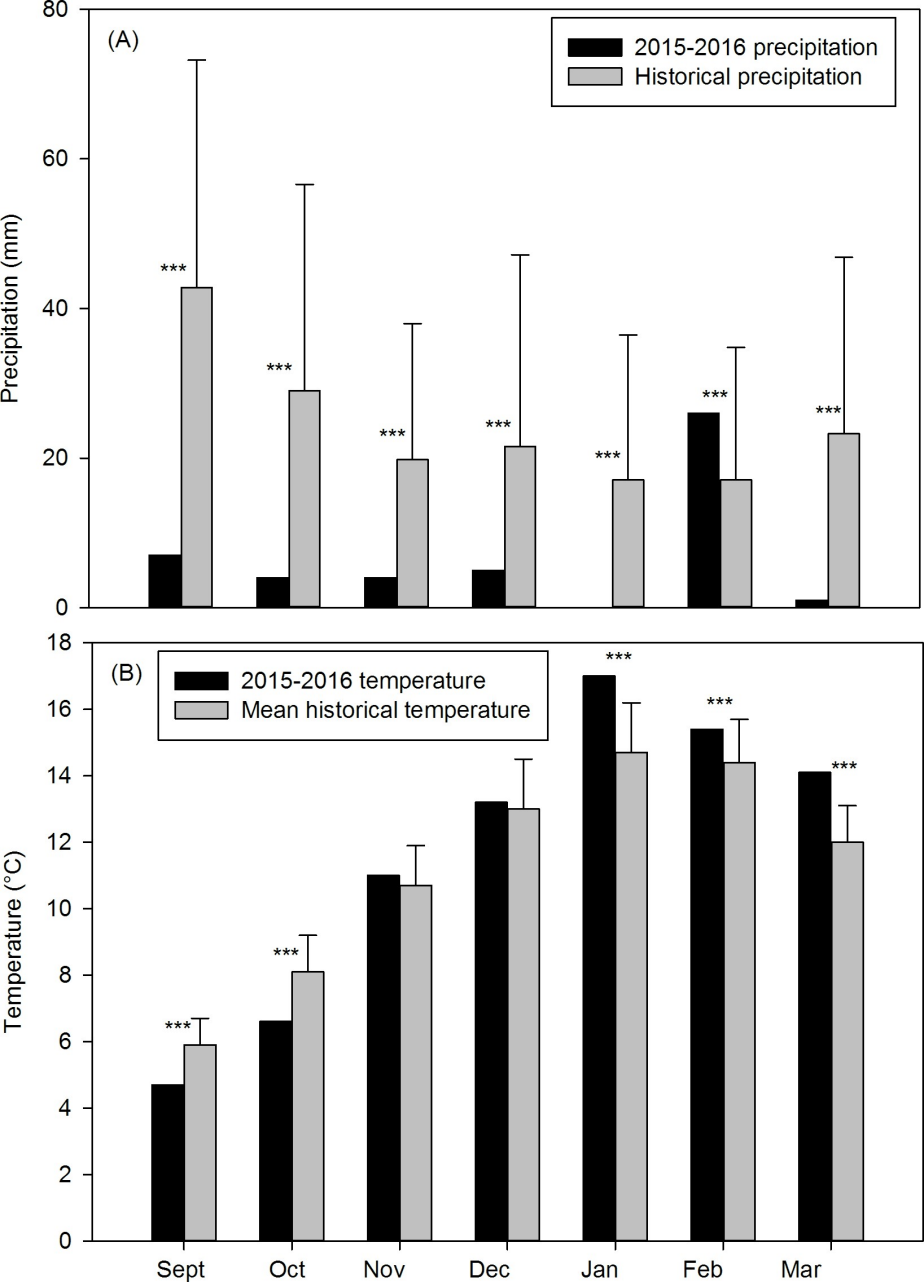
Precipitation and temperature in the 2015–2016 growing season. **(**A) Accumulated precipitation (September 2015-March 2016) and mean historical precipitation ± SD (1928–2016). (B) Mean temperature for the same period and historical mean temperature ± SD (1970–2016) (San Ramón ranch meteorological station, L. Ghermandi unpublished data). ***P< 0.001.

The spring-summer 2015–2016 seasons were dry (Fig 1). Accumulated precipitation during this period was 77% lower than the historical value (47 mm. vs. 171.6 mm, *t*_*87*_ = 17.86, *P* < 0.001) making this the driest period for the last 66 years (Fig 1). The spring-summer 2015–2016 mean temperature was higher than the historical value (11.7 ± 4.6°C vs. 11.2 ± 3.3°C, *t*_47_ = 5.06, *P* < 0.001). Mean temperatures for January, February and March (summer) were significantly higher than historical values (Fig 1).

### Species richness in the non-degraded grassland

We found 34 species in the monitored non-degraded grassland, 36 in Acaena-ND and 28 in Gap-ND microsites. Eleven of these species, including seven perennial native herbs, were exclusive to Acaena-ND microsite (eg. *Arjona tuberosa*, *Sysirinchium arenarium*, *Tristagma patagonicum*). When we compared species richness between Acaena-ND and Gap-ND microsites we found nine species (six native) growing exclusively in Acaena-ND (eg. *Hordeum comosum*, Table 2).

**Table 2:** List of species present in the microsites and the non-degraded grassland. Species present in non-degraded grassland, in *A*. *splendens* senescent plants, and in gaps in years 1999, 2000, 2001, and 2005.

Species	Acaena	Gap	Grassland
*Acaena pinnatifida* (Rosaceae)	*	*	*
*Acaena poeppigiana* (Rosaceae)		*	*
*Acaena splendens* (Rosaceae)	*	*	*
*Apera interrupta* (Poaceaea)	*	*	*
***Arjona tuberosa* (Schoepfiaceae)**	*		
*Boopis gracilis* (Calyceraceae)	*	*	*
*Bromus tectorum* (Poaceae)	*	*	*
***Camissonia dentata* (Onagraceae)**	*		
*Carduus thoermeri* (Asteraceae)	*	*	*
***Cerastium arvense* (Caryophyllaceae)**	*		
*Collomia linearis* (Polemonicaceae)	*	*	*
***Conium maculatum* (Apiaceae)**	*		
*Coniza lechleri* (Asteraceae)	*	*	*
*Draba verna* (Brassicaceae)	*	*	*
*Ephedra chilensis* (Ephedraceae)			*
*Epilobium paniculatum* (Onagraceae)	*	*	*
*Erodium cicutarium* (Geraniaceae)	*	*	*
*Euphorbia collina* (Euphorbiaceae)	*	*	*
*Festuca argentina* (Poacaeae)			*
*Festuca pallescens* (Poacaeae)	*	*	*
***Galium richardianum* (Rubiaceae)**	*		
*Heliotropium paronychioides* (Boraginaceae)		*	*
*Holcus lanatus* (Poaceae)			*
*Holosteum umbellatum* (Caryophyllaceae)	*	*	*
*Hordeum comosum* (Poaceae)	*		*
***Hypochaeris incana* (Asteraceae)**	*		
*Lactuca serriola* (Asteraceae)		*	*
*Madia sativa* (Asteraceae)			*
*Microsteris gracilis* (Polemoniaceae)	*	*	*
*Myosotis discolor* (Boraginaceae)		*	*
*Nicotiana linearis* (Solanaceae)			*
***Pappostipa humilis* (Poaceae)**	*		
*Pappostipa speciosa* (Poaceae)	*	*	*
*Plagiobothrys verrucosus* (Boraginaceae)	*	*	*
*Poa lanuginosa* (Poacaeae)	*	*	*
*Rhodophiala mendocina* (Amaryllidaceae)	*	*	*
*Rumex acetosella* (Polygonaceae)	*	*	*
*Senecio bracteolatus* (Asteraceae)	*	*	*
*Sisymbrium altissimum* (Brassicaceae)	*	*	*
***Sisyrinchium arenarium* (Iridaceae)**	*		
***Taraxacum officinale* (Asteraceae)**	*		
***Tragopogon dubius* (Asteraceae)**	*		
***Tristagma patagonicum* (Iridaceae)**	*		
*Triptilion achilleae* (Asteraceae)	*	*	*
*Vulpia australis* (Poaceae)	*	*	*
Richness	36	28	34

*Indicates presence of the species. Species highlighted in bold are exclusive to *A*. *splendens* senescent plants.

**Fig 2:**
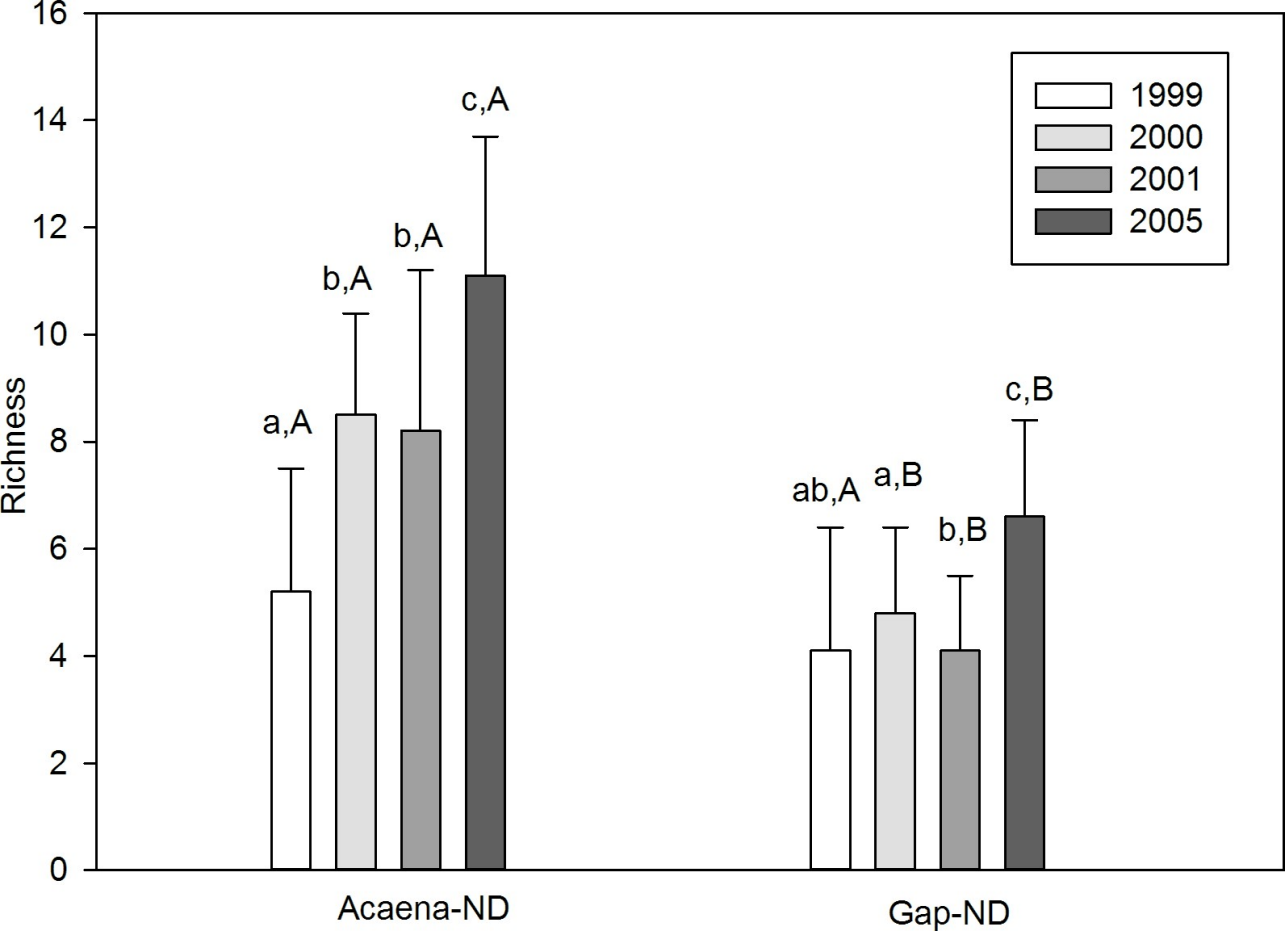
Species richness in Acaena-ND and Gap-ND microsites. Mean species richness ± SD in Acaena-ND and Gap-ND microsites (non-degraded grassland) in November 1999–2001, and 2005. Lower-case letters indicate significant differences between years in the same microsite. Capital letters indicate significant differences between microsites in the same year.

Species richness was higher in Acaena-ND than in Gap-ND (2000: *U* = 12, *P*< 0.001; 2001: *U* = 17.5, *P* < 0.001; 2005: *U* = 5, *P* < 0.001), with the exception of November 1999 (*U* = 69.5, *P* = 0.32) (Fig 2). Comparing years, richness was higher in 2005 in both microsites (Acaena-ND: χ2 = 18.8, *df* = 3, *P* <0.001, *n* = 10; Gap-ND: χ2 = 27, *df* = 3, *P* < 0.001, *n* = 18) (Fig 2). The number of exclusive species was always higher in Acaena-ND than in Gap-ND (S1 Table).

### Functional groups in the non-degraded grassland

**Fig 3:**
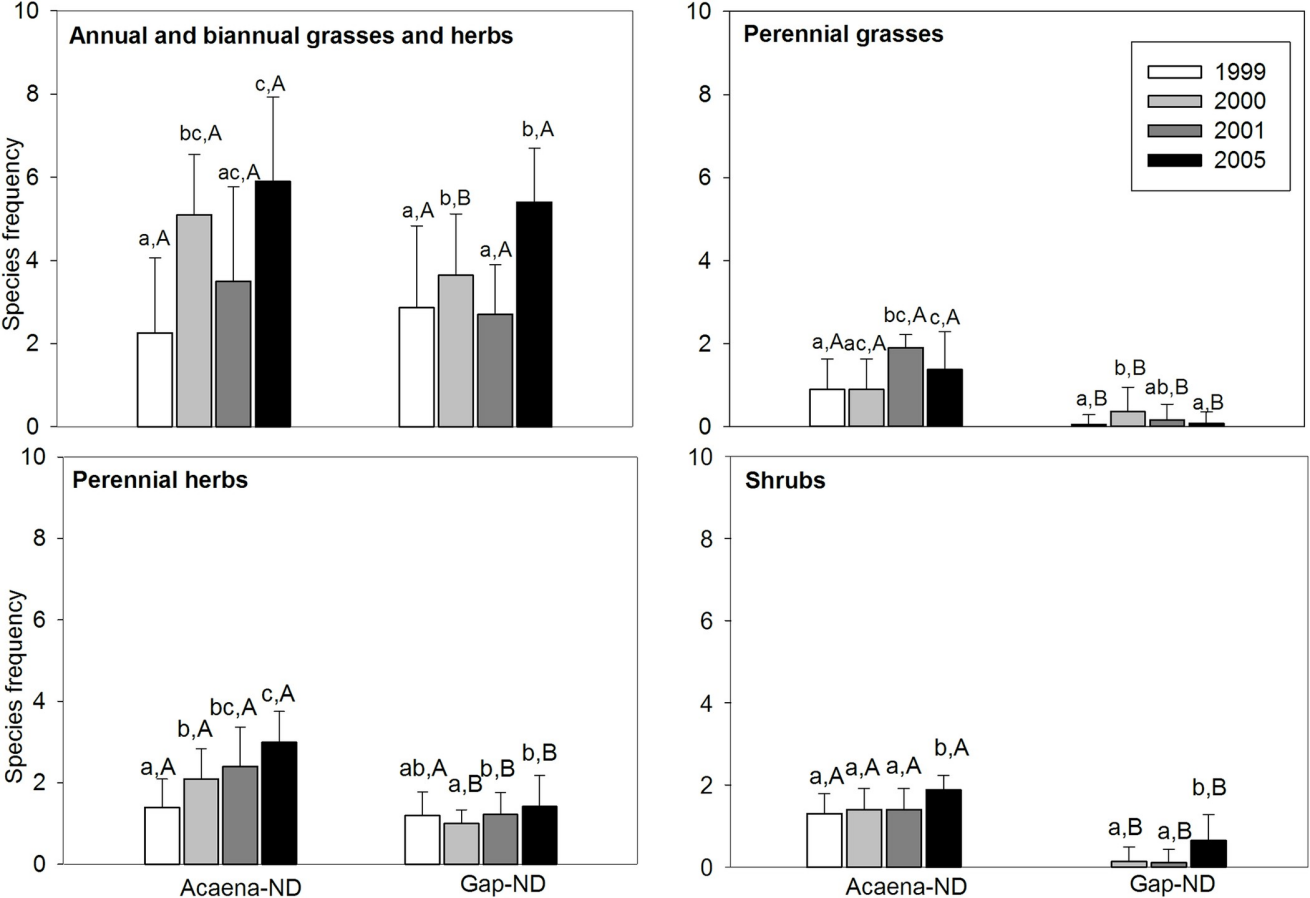
Functional groups in Acaena-ND and Gap-ND microsites. Mean frequency of species ± SD grouped in functional groups in Acaena-ND and Gap-ND in non-degraded grassland (November 1999, 2000, 2001 and 2005). Lower-case letters indicate significant differences of each functional group between years in the same microsite. Capital letters represent significant differences between microsites in the same year.

In Acaena-ND and Gap-ND microsites the annual and biannual herbs and annual grasses functional group (AHG) dominated in all years. The frequency of the AHG functional group was higher in Acaena-ND than in Gap-ND in 2000 (*U* = 38.5, *P* = 0.01), and was higher than the other functional groups in 2000 and 2005 in both microsites (Acaena-ND: χ2 = 19.21, *df* = 3, *P* < 0.001, *n* = 8; Gap-ND: χ2 = 30.33, *df* = 3, *P*< 0.001, *n* = 18) (Fig 3). The frequency of the PG functional group was always higher in Acaena-ND than in Gap-ND (1999: *U* = 30, *P* < 0.001; 2000: *U* = 50.5, *P*< 0.04; 2001: *U* = 0.5, *P* < 0.001; 2005: *U* = 21, *P* = 0.001) (Fig 3). The frequency of the PH functional group increased significantly over the years in Acaena-ND (χ2 = 11.33, *df* = 3, *P* = 0.01, *n* = 10) and it was higher than Gap-ND in 2000, 2001 and 2005 (2000: *U* = 19, *P* < 0.001; 2001: *U* = 28.5, *P* = 0.002; 2005: *U* = 8, *P* < 0.001). The frequency of the SHR functional group was higher in Acaena-ND than in Gap-ND (2000: *U* = 15, *P* < 0.001; 2001: *U* = 12, *P* < 0.001; 2005: *U* = 4.5, *P* < 0.001), and was the highest in 2005 in both microsites (Acaena-ND: χ2 = 12; *df* = 3; *P* = 0.007, *n* = 10; Gap-ND: χ2 = 15.3; *df* = 2; *P* < 0.001, *n* = 18). The shrub functional group was absent in Gap-ND in 1999 (Fig 3).

### *Acaena splendens* seedling emergence in the non-degraded grassland

**Fig 4:**
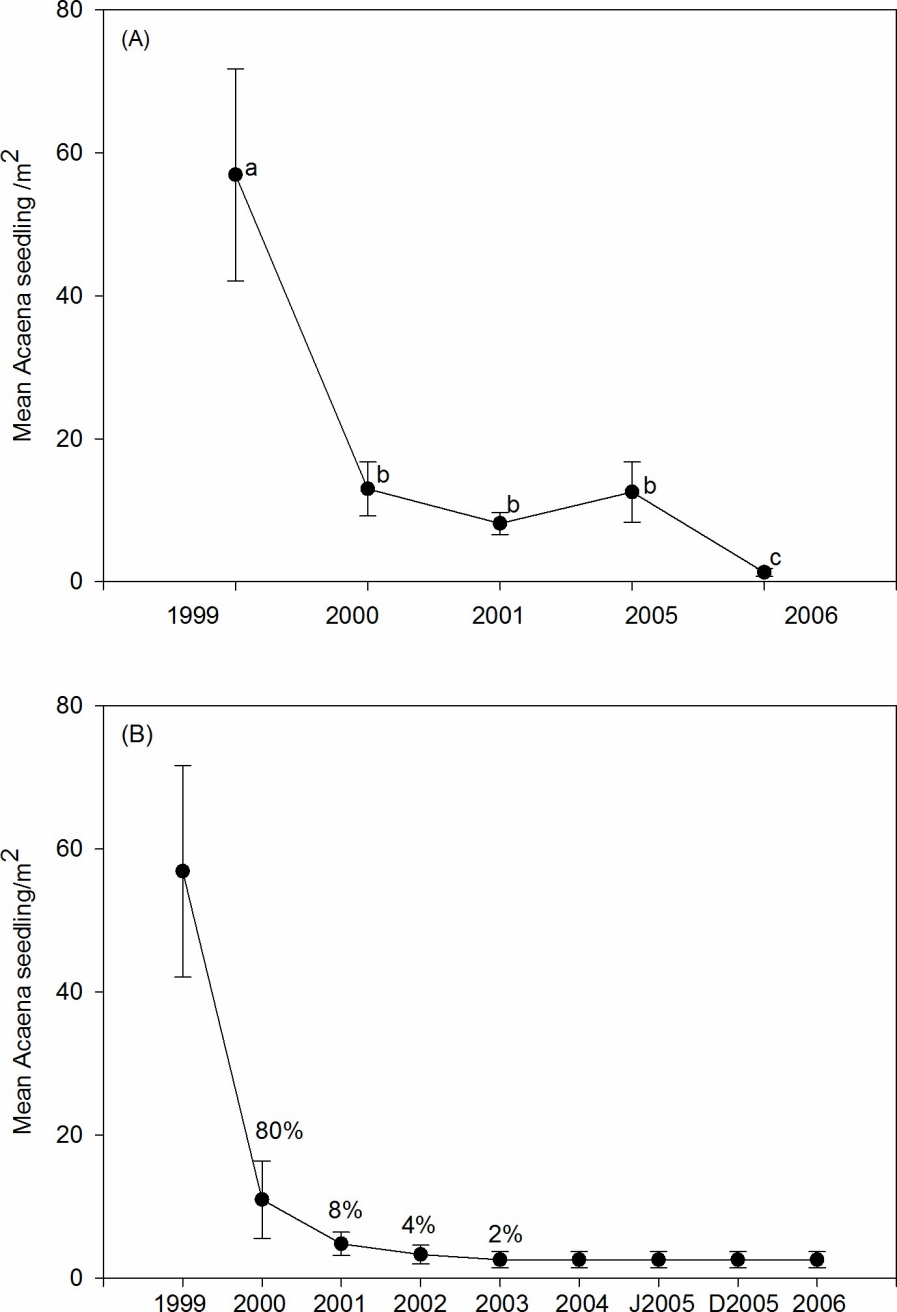
*Acaena splendens* seedling emergence and mortality. Mean seedling.m^-2^ of *A*. *splendens* (A) emerged from 1999 to 2006, (B) from the cohort that emerged in 1999 and was monitored until 2006. We indicated percentage mortality on each sampling date.

In the non-degraded grassland, *A*. *splendens* seedling emergence differed between years (χ2 = 16.9; *df* = 4; *P* = 0.002, *n* = 10) (Fig 4). The highest number was recorded in 1999 (56.9 ± 14.8 seedling.m^-2^) (*P* <0.05) and the lowest in 2006 (0.7 ± 0.6 seedling.m^-2^) (*P* <0.05). Emergence was similar in 2000, 2001 and 2005 (2000 vs. 2001: *P* < 0.06, *Z* = -1.83; 2000 vs. 2005: *P* <0.72, *Z* = -0.35; 2001 vs. 2005: *P* < 0.23, *Z* = -1.19). *A*. *splendens* seedling mortality was 80% in 2000, 8% in 2001, 4% in 2002 and 2% in 2003. At the last monitoring (2006), 2.6.m^-2^ seedlings survived (Fig 4).

### *Festuca pallescens* seedling emergence in the degraded grassland

In degraded grassland in May 2015 (fall, one month after sowing), 0.75% *F*. *pallescens* seeds germinated in Acaena-D and Gap-D microsites. In September 2015 (spring, five months after sowing) 38.5% germinated in Acaena-D and 8% in Gap-D. Mean seedling density was significantly higher in Acaena-D (15.7± 1.7 seedling.m^-2^) than in Gap-D (3.4 ± 0.9 seedling.m^-2^) (*Z* = -3.86; *P*< 0.001). In both microsites, most of the seedlings had two leaves, but we found a higher number of seedlings with two leaves in Acaena-D (82%) than in Gap-D (78%) (Z = -3.8; *P* < 0.001). We found a higher percentage of seedlings with exposed radicles in Gap-D (33%) than in Acaena-D (15%), although we did not find significant differences (Z = -0.6; *P* = 0.55). Seedling mortality was higher in Gap-D than in Acaena-D (97% *vs*. 88%, *Z* = -2.6; *P* = 0.007) in late spring (December 2015), mean seedling density being very low in both microsites (Acaena-D: 1.9 ± 0.6; Gap-D: 0.1 ± 0.1). In March 2016 (early fall), seedling mortality was 100% in both microsites.

### Environmental variables in the degraded grassland

**Fig 5:**
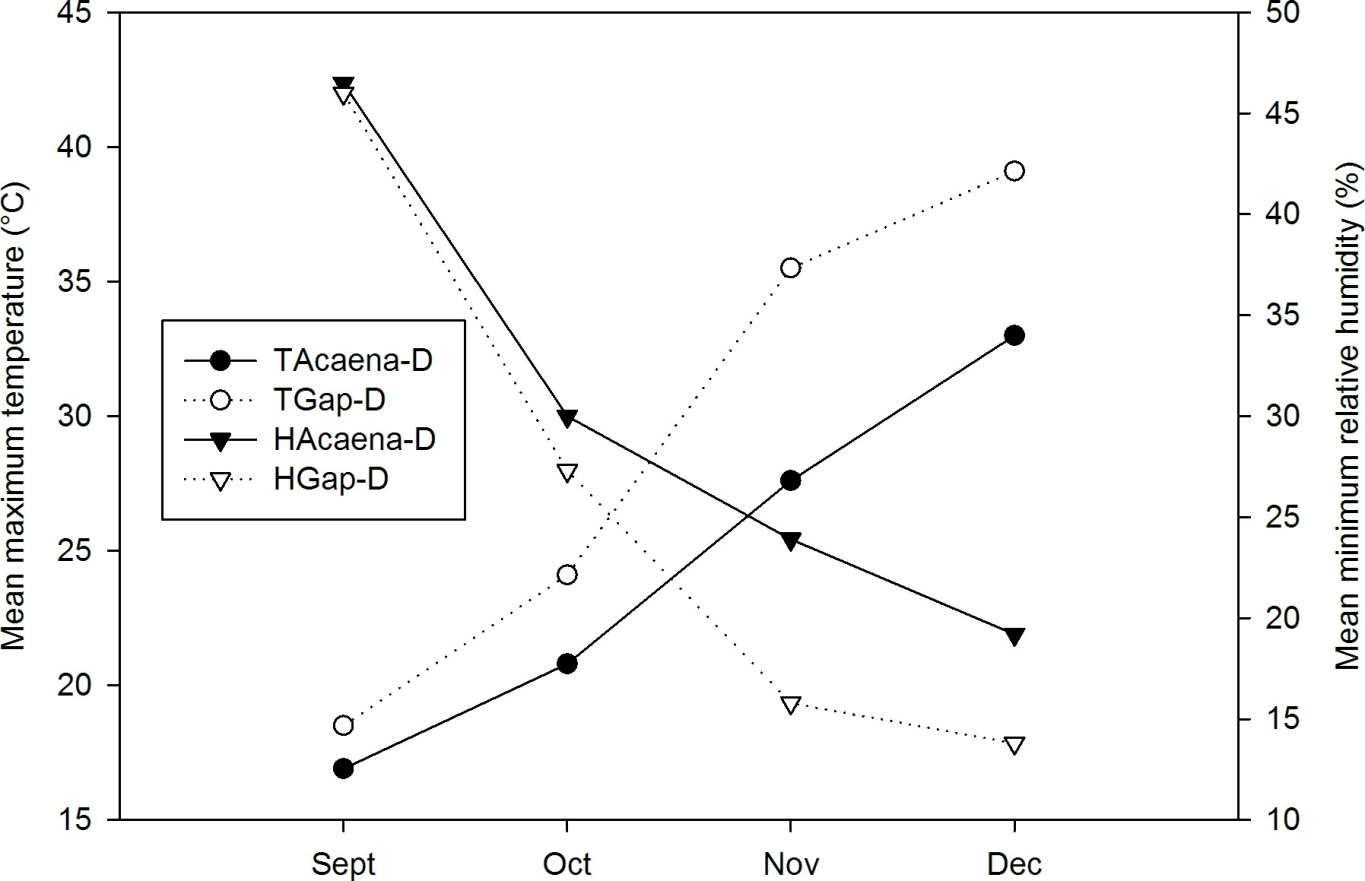
Temperature and relative humidity in the microsites of degraded grassland. Mean maximum temperature (T) and minimum relative humidity (H) recorded by dataloggers in Acaena-D and Gap-D microsites from September to December 2015.

In the degraded grassland, the mean maximum temperature was always higher in Gap-D than in Acaena-D (*P* < 0.05) (Fig 5). The highest mean maximum temperature was recorded in December in Gap-D (39.1±5°C). In September, in Gap-D 38.5% of the temperature records were between 20 and 30°C. In November 75% of the temperature records were above 30°C in Gap-D (30% of which were between 40 and 50°C), and 36% in Acaena-D (3% between 40 and 50°C) (χ2 = 74; *df* = 10; *P* < 0.001) (Fig 5, S1 Fig). In December, the temperature in Gap-D was always above 30°C (37% between 40 and 50°C), whereas only 50% were above 30°C in Acaena-D (S1 Fig). The mean minimum humidity was always lower in Gap-D than in Acaena-D (*P* < 0.05), except for September (*P* > 0.05). Humidity abruptly decreased in October in both microsites and the lowest mean minimum humidity was found in December in Gap-D (13.8 ± 3.4) (Fig 5). Humidity values for November and December were always below 50%. In Gap-D, this decrease was remarkable, with extreme values of < 10% recorded since October. In contrast, values < 10% were never recorded in Acaena-D (S1 Fig).

With respect to soil variables, total C, N and the C:N ratio were higher in Acaena-D than in Gap-D (C: 1.6 ± 0.1% *vs*.1 ± 0.1%; *t*_*4*_ = -4.25, *P* = 0.013; N: 0.1 ± 0.01% *vs*. 0.07 ± 0.01%; t_*4*_ = -3.30, *P* = 0.030; and C:N 16.3 ± 0.6 *vs*. 14.6 ± 0.07; *t*_*4*_ = -2.50; *P* = 0.001, respectively). The organic matter value was also higher in Acaena-D (2.75%) than in Gap-D (1%).

## Discussion

In arid and semiarid environments plant recruitment is water limited [45], and facilitation by nurse shrubs creates safe sites for seedling establishment. Nevertheless, facilitation fails under extremely stressful conditions [46, 47]. We found that senescent plants of the dwarf shrub *A*. *splendens* maintained grassland richness and facilitated the seedling recruitment of a palatable grass in semiarid Northwestern Patagonia. We also found the threshold at which the facilitation process ceases to be effective.

The presence of nurse plants in a community results in different compositions of species growing beneath and outside nurses, and can increase species richness [21]. *A*. *splendens* shrubs promoted the seedling recruitment of exclusive native species in the non-degraded grassland. Some of these species (eg. *Arjona tuberosa*, *Tristagma patagonicum*) established exclusively in Acaena microsite increasing the community richness. Also, we highlight that species richness was always higher in Acaena, including in the 1999 dry spring that was preceded by a strong La Niña event [48] suggesting that even in unfavorable conditions *A*. *splendens* contributes to the maintenance of grassland diversity.

The *A*. *splendens* shrubs facilitated the establishment of seedlings of three functional groups in the non-degraded grassland; perennial grasses, perennial herbs, and shrubs, all of which are also present in gaps, but in lower proportions. In contrast, annual and biannual grass and herbs are more stress-tolerant than the seedlings of perennial grasses and shrubs [32, 48], and thus were found in the same proportions in both microsites. Annual and biannual grasses and herbs are typical gap species that complete their life cycle from autumn to spring [48]. Seedlings of perennial grasses and shrubs also recruit in gaps, but severe water deficit strongly affect their survival [32]. In the case of the dominant perennial grasses recruitment is very important as it guarantees the matrix turnover; in effect, tussock grasses are long-lived, but not eternal. Similarly, in other Patagonian degraded grasslands [49] found higher grass seedling density underneath shrubs than in bare soil. The *A*. *splendens* nurse plants were clearly safe sites for the establishment of native perennial herbs (eg. *Arjona tuberosa*, *Sisyrinchium arenarium*) which were present exclusively in this microsite. Also, *A*. *splendens* promoted the establishment of other shrubs in dry years, since this functional group was absent in gaps in spring of 1999. In our study we demonstrated that facilitation not only influences grassland diversity, but also the proportion of functional groups. Also the nurse shrub *Potentilla fruticosa* (grassland of NW China) changed the proportion of functional groups [50]. This study showed that legumes and graminoids were more strongly facilitated than forbs, highlighting the importance of these results at community level.

Senescent *A*. *splendens* plants facilitate self-replacement. Although the species produces seeds that are principally dispersed by zoochory because of their spiny surface, many seeds fall and germinate inside the mother plants. Seedlings mainly recruit in the rainy season, and the high recruitment recorded in *A*. *splendens* nurse plants was probably related to the exceptionally late 1999 winter conditions. The August rainfall and temperature were higher than the historical value plus the standard deviation (181.3 *vs* 75.4± 47.6 mm; 5°C *vs* 3.7°± 1.2°C). It is known that small variations in temperature play an important role in plant recruitment. In the same study area and year, we recorded abundant recruitment of another two native shrubs, *Senecio bracteolatus* [51] and *Fabiana imbricata* [52]. The drought of summer 2000 limited soil water, causing high mortality of *A*. *splendens* seedlings, although it is likely that self-thinning was other important factor, due to the high seedling density. Although seedling mortality was high, 2.6 plants.m^-2^ survived after seven years and this density guarantees self-replacement and rejuvenation of the *A*. *splendens* population.

Shrubs are widely used as nurse plants in restoration programs [10, 46]. We evaluated whether an unpalatable shrub can facilitate a grass with high forage value in the degraded grassland. The abundant presence of the palatable *F*. *pallescens* is required since ranch managers in Northwestern Patagonia use the grassland for stockbreeding, without the addition of extra forage. In our study the *A*. *splendens* nurse plants facilitated *F*. *pallescens* seedling recruitment by reducing physical stress and increasing nutrients in the degraded grassland. For instance, the temperature in Acaena-D was 10°C lower than Gap-D (with a maximum difference of 24°C) during the critical spring-summer months. Plant canopies can buffer daily temperature fluctuations and can intercept and condense water from the air [53]. This effect favors seed imbibition and germination, and increases seedling survival [54].

Air humidity decreased rapidly from October to December in both microsites following the rainfall pattern, but the driest days were more frequent in gaps. Although we did not measure soil moisture, we observed moister soil in *A*. *splendens* shrubs during monitoring. Experiments using isotope analysis have shown that woody plants can transport water from deep soil layers to the surface at night (hydraulic lift) [55]. *Artemisia tridentata* improves the soil moisture of neighboring plants through this process [56] and it is possible that the soil near *A*. *splendens* plants was moister due to the same mechanism. Another factor that contributes to increasing soil moisture near the nurse plants is the amount of organic matter, which retains more water than sandy soil [25].

Organic matter influences soil-water relations by increasing soil aggregation and water-holding capacity [25]. The soils in our study area are Haploxerolls with a surface layer (mollic epipedon) containing 2% organic matter [42]. In *A*. *splendens* nurse plants we found more organic matter (2.7%) than in the Gaps, confirming that this shrub improves soil fertility [57]. In semiarid environments, C availability is important, as a low percentage of C limits microbial activity. The estimated C:N ratios (14 in Acaena-D and 16 in Gap-D) are considered values that allow N mineralization and availability [57, 58].

The expansion and contraction of soil caused by freezing exposes and breaks seedling roots, causing seedling death [59]. Depth of frost penetration is usually greater in sandy soils like Patagonian steppe soil [25]. In Patagonian grasslands, the emergence of *F*. *pallescens* seedlings in months of highest frequency of frost heaving negatively influenced recruitment in gaps [32]. In our study, *A*. *splendens* nurse plants organic matter moderates soil temperature, which decreases the severity of frost heaving protecting *F*. *pallescens* seedlings.

*Festuca pallescens* recruited easily but suffered high mortality in late summer. The high seedling mortality was probably due to the unusually dry and hot spring, which was the driest for 66 years, with precipitations of 20 mm, 93 mm lower than the historic value. In the non-degraded grassland, where the vegetation cover has been monitored in fixed plots since 1999, the 2015 cover was significantly lower than in the previous 16 years (L. Ghermandi unpublished data). Other study showed that summer drought caused seedling mortality of *F*. *pallescens* [34]. In Patagonian grassland located in the Chubut Province, *F*. *pallescens* became successfully established exclusively in wet summers [32, 60]. Our findings showed that the *A*. *splendens* facilitation of *F*. *pallescens* recruitment collapsed under extreme water stress conditions. Similarly, in other study tussocks facilitated seedling establishment of herbaceous species, but the effect declined in extreme drought [14]. In this circumstance, water availability becomes more important than nurse protection, and there is a switch from facilitation to competition [61]. These results reinforce the idea that plant-plant interactions are primarily mediated by plant effects on micro-environmental stress and resource availability, but are ultimately controlled by external factors [62]. However, we consider important to remark that *A*. *splendens* microsites provided better conditions for the establishment and survival of *F*. *pallesccens* seedlings until December (late spring) than gaps despite the adverse climatic conditions.

The abundance of *A*. *splendens* is high in overgrazed sites. However our results provide evidences of the facilitation of *F*. *pallescens* by senescent plants of *Acaena splendens*. The *A*. *splendens* plants are advantaged compared to others nurse plants due to the progressive loss of competitivity [63]. When they are dying contribute with organic matter to soil. At the community level this implies a beneficial replacement of species, from an undesirable shrub to economically important species. However, the employment of *A*. *splendens* as nurse plants in restoration programs probably must be accompanied by the exclusion of livestock the first years until the forage grasses reach sexual maturity. Further, given that in arid and semiarid environments precipitations are very variable in time [64], probably during the first years after sowing the rainfalls must be observed and, eventually, they must be complemented with irrigation pulses to facilitate the grasses recruitment. In this respect, mores studies are needed.

Further research should investigate ways to overcome water limitation in the restoration programs. Ecophysiology studies should identify water-stress tolerant species to facilitate their re-introduction, thus improving grassland productivity. Restoration researchers should test other techniques, such as seedling and adult planting, or the utilization of hydrogel in planting to improve the water status of seedlings. These techniques have given good results in the arid Monte, a shrubby environment of Patagonia [65].

Woody plants are often seen as indicators of land degradation, whereas they provide a benign microclimate that facilitates the regeneration and growth of their own offspring as well as other plants [66]. Future research could continue to explore the importance of shrubs as nurse plants in the regulation of diversity in grassland communities, an aspect especially important in the conservation and functioning of these vulnerable ecosystems.

## Supporting information

S1 Fig Frequency of maximum temperature and minimum humidity in the microsites of degraded grassland. (PDF)

S1 Table List of species in *Acaena splendens* nurse plants and in gaps. (PDF)
