# Supplementary material for: Dwarf shrub facilitates seedling recruitment and plant diversity in semiarid grasslands
Source: PLoS One. 2019 Feb 7;14(2):e0212058. doi: 10.1371/journal.pone.0212058 (PMC6366867; doi:10.1371/journal.pone.0212058)
Supplement: S1 Fig — (PDF) [file pone.0212058.s001.PDF]

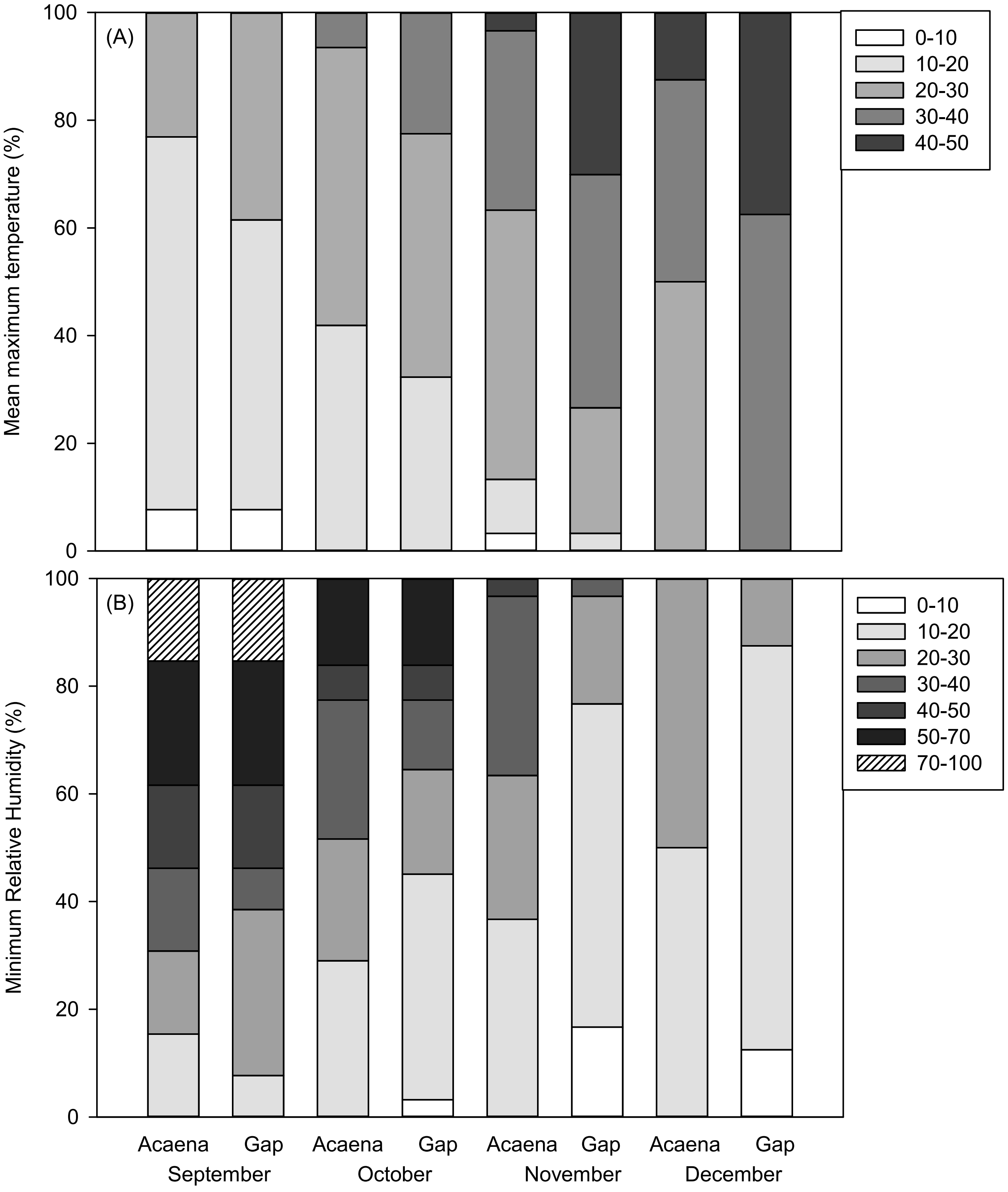

S1 Fig. Frequency of (A) mean maximum temperature (°C), and (B) mean minimum humidity (%) in Acaena-D and Gap-D during spring 2015. Values are grouped in ranges.
