# Supplementary material for: Dwarf shrub facilitates seedling recruitment and plant diversity in semiarid grasslands
Source: PLoS One. 2019 Feb 7;14(2):e0212058. doi: 10.1371/journal.pone.0212058 (PMC6366867; doi:10.1371/journal.pone.0212058)
Supplement: S1 Table — (PDF) [file pone.0212058.s002.pdf]

S1 Table. List of species (family) in *Acaena splendens* nurse plants and in gaps 1999, 2000, 2001, and 2005 years in non-disturbed grassland. Total richness and exclusive species.

Species	1999		2000		2001		2005	
**Annual and bianual grasses and herbs**	Acaena	Gap	Acaena	Gap	Acaena	Gap	Acaena	Gap
Apera interrupta (Poacaeae)**	*	*	*	*	*	*	*	*
Boopis gracilis (Calyceraceae)	*				*	*	*	*
Bromus tectorum (Poaceae)**	*		*		*		*	*
Camissonia dentata(Onagraceae)							*	
Carduus thoermeri (Asteraceae)**	*	*	*	*	*	*	*	*
Collomia linearis (Polemoniaceae)	*	*	*	*				
Draba verna (Brassicaceae)**	*	*	*	*	*	*	*	*
Erodium cicutarium (Geraniaceae)**	*	*	*	*	*	*	*	*
Epilobium paniculatum (Onagraceae)**					*	*	*	*
Heliotropium paronychioides (Boraginaceae)		*						
Holosteum umbellatum (Caryophyllaceae)**	*	*	*	*	*	*	*	*
Microsteris gracilis (Polemoniaceae)	*			*		*	*	*
Myosotis discolor (Boraginaceae)**				*				*
Plagiobothrys verrucosus (Boraginaceae)	*	*	*	*	*	*	*	*
Sisimbrium altissimum (Brassicaceae)**	*	*	*			*	*	*
Triptilion achilleae (Asteraceae)		*	*	*	*	*	*	*
Tragopogon dubius (Asteraceae)**							*	
Vulpia australis (Poaceae)	*	*	*	*	*	*	*	*
**Perennial herbs**								
Acaena pinnatifida (Rosaceae)		*	*		*	*	*	*
Acaena poepiggiana (Rosaceae)				*				
Arjona tuberosa (Schoepfiaceae)							*	
Cerastium arvense (Caryophyllaceae)**			*		*		*	

Coniza lechleri (Asteraceae)							*	*
Conium maculatum (Apiaceae)					*			
Euphorbia collina (Euphorbiaceae)	*		*	*	*	*	*	*
Galium richardianum (Rubiaceae)			*					
Hypochaeris incana (Asteraceae)							*	
Lactuca serriola (Asteraceae)**						*		
Rhodophiala mendocina (Amaryllidaceae)	*	*	*		*	*	*	*
Rumex acetosella (Polygonaceae)**	*	*	*	*	*	*	*	*
Sisyrinchium arenarium (Iridaceae)	*		*		*		*	
Taraxacum officinale (Asteraceae)**	*		*					
Tristagma patagonicum (Iridaceae)					*			
**Perennial grasses**								
Festuca pallescens (Poaceae)				*	*	*	*	*
Hordeum comossum (Poaceae)	*						*	
Poa lanuginosa (Poaceae)	*	*	*	*	*	*	*	*
Pappostipa speciosa (Poaceae)	*		*	*	*	*	*	
Pappostipa humilis (Poaceae)	*		*					
**Shrubs**								
Acaena splendens (Rosaceae)	*		*	*	*	*	*	*
Senecio bracteolatus (Asteraceae)	*		*	*	*	*	*	*
**Total richness**	23	15	23	19	25	21	30	23
**Exclusive species**	11	3	9	5	6	2	8	1

** indicates exotic species.
